# Intracerebral delivery of Carboplatin in combination with either 6 MV Photons or monoenergetic synchrotron X-rays are equally efficacious for treatment of the F98 rat glioma

**DOI:** 10.1186/1756-9966-31-78

**Published:** 2012-09-20

**Authors:** Laure Bobyk, Magali Edouard, Pierre Deman, Julia Rousseau, Jean-François Adam, Jean-Luc Ravanat, François Estève, Jacques Balosso, Rolf F Barth, Hélène Elleaume

**Affiliations:** 1 INSERM U836 Équipe 6, Grenoble Institut des Neurosciences, Grenoble, France; 2 Laboratoire "Lésions des Acides Nucléiques", SCIB-UMR-E n°3 (CEA/UJF) Institut Nanosciences et Cryogénie, CEA/Grenoble, Grenoble, France; 3 European Synchrotron Radiation Facility, Medical Beamline ID17, Grenoble, France; 4 Department of Pathology, The Ohio State University, Columbus, Ohio, 43210, USA; 5 Université Joseph Fourier; Centre Hospitalier Universitaire, Grenoble, France

**Keywords:** F98 rat glioma, Carboplatin, Osmotic pumps, Intracerebral delivery, Radiotherapy with 6 MV photons or synchrotron X-rays

## Abstract

**Background:**

The purpose of the present study was to compare side-by-side the therapeutic efficacy of a 6-day infusion of carboplatin, followed by X-irradiation with either 6 MV photons or synchrotron X-rays, tuned above the K-edge of Pt, for treatment of F98 glioma bearing rats.

**Methods:**

Carboplatin was administered intracerebrally (i.c.) to F98 glioma bearing rats over 6 days using Alzet^TM^ osmotic pumps starting 7 days after tumor implantation. Radiotherapy was delivered in a single 15 Gy fraction on day 14 using a conventional 6 MV linear accelerator (LINAC) or 78.8 keV synchrotron X-rays.

**Results:**

Untreated control animals had a median survival time (MeST) of 33 days. Animals that received either carboplatin alone or irradiation alone with either 78.8 keV or 6 MV had a MeSTs 38 and 33 days, respectively. Animals that received carboplatin in combination with X-irradiation had a MeST of > 180 days with a 55% cure rate, irrespective of whether they were irradiated with either 78.8 KeV synchrotron X-rays or 6MV photons.

**Conclusions:**

These studies have conclusively demonstrated the equivalency of i.c. delivery of carboplatin in combination with X-irradiation with either 6 MV photons or synchrotron X-rays.

## Background

High Z-enhanced synchrotron stereotactic radiotherapy relies on the dose-enhancement obtained when tumors, previously filled with a high-Z elements, are irradiated with medium energy x-rays (50–100 keV) in stereotactic conditions. The concept comes initially from the observation in the late 70’s, of additional blood damages in pediatric diagnostic radiology, when using contrast agents [1]. The use of medium energy x-rays to treat cancer could appear surprising nowadays, specially for brain tumors, but as the photoelectric cross section increases proportionally to Z^4^/E^3^ (where Z is the atomic number of matter and E the energy of photons), there is a subsequent increase of the absorbing properties restricted to the target level, due to the release of secondary particles (photoelectrons, characteristic x-rays and Auger electrons), which deposit most of the initial photon energy in the close vicinity of the primary interaction. Photoelectric effect is the photon interaction that deposits locally the largest part of the photon initial energy (when compared to coherent or incoherent scattering events). This leads to improved dose distributions in comparison with conventional high energy treatments. Numerous studies have been performed for establishing that this method meets dosimetry criteria for patients [2-8]. From 50 to 80 keV, the brain half value layer increases from 2.93 to 3.64 cm. Although these values are relatively small, the dose is increased by (i) the irradiation geometry and (ii) by the presence of sufficient amount of high Z elements inside the tumor volume (≈ 3–10 mg/mL). LINAC spectra extend from MV to kV energies, however, the contribution of kV radiation in the dose-enhancement is negligible, as shown with Monte carlo simulations or experimentally using gel dosimetry [2-5,9]. The use of a modified conventional CT scanner, such as proposed by Norman and co-workers [10], appeared to be the main limitation of the technique due to (i) the beam shape (fan or cone-shaped beam with a high aperture) which broadens the isodoses as the beam goes through the patient; (ii) a low photon flux and a high thermal load which impose long irra-diation times and cooling phases; and (iii) a broad polychromatic spectrum of the beam which increases the dose to the skull and minimizes the dose enhancement effect. For these reasons, nearly parallel intense and tunable monochromatic beams provided by synchrotron source appear to be a must for this radiotherapy technique.

In a series of publications [11-14] we have reported on the therapeutic efficacy of short-term intracerebral (i.c.) convection enhanced delivery (CED) of either carboplatin or cisplatin or alternatively prolonged intratumoral (i.t.) infusion of carboplatin either alone or in combi-nation with X-irradiation for the treatment of the F98 rat glioma [11-13]. Irradiations were carried out at the European Synchrotron Radiation Facility (ESRF) using 78.8 keV synchrotron X-rays or 6 MV photons, by a medical linear accelerator (LINAC), at the University Hospital of Grenoble, France. Carboplatin was selected for these studies because we previously have shown that it was highly effective in treating F98 glioma bearing rats [11-14]*.* However, platinum containing drugs have their limitations [15-17] for the treatment of brain tumors. These include inadequate dose-limiting toxicity and reduced uptake by brain tumors following systemic administration due to the blood brain barrier (BBB) [18]. Delivery of carboplatin by CED was well tolerated when delivered i.c. to F98 glioma bearing rats [11,12,14,19-21] and non-human primates [22] and resulted in prolonged survival and cures of the former.

Cure rates of 20% to 55% were obtained in F98 glioma bearing rats treated with prolonged infusions of either carboplatin or cisplatin using Alzet osmotic pumps alone or in combination with synchrotron X-irradiation. In these studies, the beam energy was tuned at 78.8 keV, which was just above the K-edge of Pt [12,23]. The first study carried out at the ESRF with cisplatin [23], employed synchrotron X-rays and it was hypothesized that therapeutic efficacy was dependent upon the production of Auger electrons and photoelectrons following irradiation of Pt atoms with monochromatic X-rays. Above the Pt K-edge energy (78.4 keV), extraction of electrons from the K-shell by the photoelectric effect results in the creation of vacancies. The resulting gaps are filled successively by radiative (96%) and non-radiative (4%) transitions from outer shells, thereby resulting in the release of several low energy photons and electrons. If the Pt atoms are located near or within DNA, the emitted low energy electrons can be highly destructive for DNA and lethal to tumor cells [24], even with small concentrations of Pt. We further hypothesized that drugs such as cisplatin or carboplatin were ideal candidates for this approach, since they had two advantages: *first*, they were DNA alkylating agents [25] and *second*, they were efficient carriers of Pt, which has a high Z number. The photoelectric cross-section of Pt at 78.8 keV is 2860 barns/atom, 4.8 times greater than at 78.0 keV. Therefore, if therapeutic efficacy were related to the emission of Auger electrons and photoelectrons from the Pt atoms, a greater therapeutic gain should have been observed with X-irradiation above rather than below the Pt K-edge. Furthermore, and most impor-tantly, almost no enhancement should be observed with 6 MV photons. This is because the Pt photoelectric cross is < 1 barn/atom above 1 MeV (i.e. >2860 × less than the Pt cross section above its K-edge), which is not significantly different from that of water. The Compton interaction process is dominant in this energy range and does not induce local energy deposition, contrary to the photoelectric effect. However, our studies carried out over the past 4 years have called this interpretation into question. The best survival data and cure rate (55%) ever reported with F98 glioma model were obtained by combining i.c. administration of carboplatin by means of Alzet osmotic pumps followed by synchrotron X-irradiation tuned at 78.8 keV. Therefore, it was important to carry out another study under similar conditions with 6 MV X-rays using a LINAC instead of synchrotron radiation to demonstrate that this effect was independent of the X-ray source. In the present study we have shown the equivalency of synchrotron X-rays [11,26] and 6 MV photons in combination with prolonged i.c. administration of carboplatin to produce prolonged survivals and cures of F98 glioma bearing rats.

## Methods

All operative procedures related to animal care strictly conformed to the Guidelines of the French Government (licenses #380324 and #A3818510002). The protocol was approved by the Grenoble Institute of Neurosciences Ethical Committee (H. Elleaume, PhD, permit #381026). Experiments were performed under anesthesia, and every effort was made to minimize the number of animals used and to alleviate pain and suffering during the experimental procedures.

### Tumor model

F98 rat glioma cells (American Type Culture Collection #CRL-2397) were cultured in Dulbecco’s modified eagle’s medium (DMEM, Invitrogen, France), supplemented with 10% fetal bovine serum and 1% penicillin/streptomycin. For tumor cell implantation, male Fischer rats (Charles River Laboratory, L’Abresles, France), weighing 260–310 g, were anesthetized with isoflurane, followed by an i.p. injection of ketamine (60 mg/kg body weight (b.w.) and xylazine, 7 mg/kg (b.w.). The animals’ eyes were coated with an ocular lubricant prior to surgery to prevent the development of keratitis. The rats were placed in a stereotactic headframe (David Kopf Instruments, Tujunga, California) and a Hamilton syringe (#701 N 10μL) was mounted on it and this was attached to a syringe pump to control the injection rate. A skin incision was made, the scalp was reflected and a burr hole was drilled 3.5 mm to the right of the bregma. A 4 μL suspension of 1000 F98 glioma cells in serum-free DMEM was injected stereotactically into the right caudate nucleus of syngeneic Fischer rats using a syringe pump (KDS310; Geneq, Inc., Montréal, Quebec, Canada). The cells were injected over 8 min via a 26 gauge needle, which was inserted to a depth of 7 mm from the skull surface and then withdrawing it to the target depth of 6.5 mm. After tumor cell implantation, the needle was left in place for 2 min and then slowly withdrawn. The burr hole in the calvarium was sealed with bone wax, and the operative field was cleansed with povidone iodine before closure of the scalp incision by sutures.

### Intracerebral delivery of carboplatin and experimental plan

Carboplatin (M.W = 371.25 Da, Faulding Pharmaceuticals, Asnières, France) was diluted in 5% dextrose to obtain a final concentration of 0.5 mg/mL. ALZET osmotic pumps (model #2001, Charles Rivers Laboratories, L’Abresles, France) and brain infusion kits (Bilaney, Dusseldorf, Germany) were assembled and filled with carboplatin. The pumps were stored in the dark in a sterile solution of 0.9% saline at 37°C for 24 h prior to their use. Seven days after tumor cell implantation the animals were anesthetized and the scalp incision was re-opened. The bone wax was removed with a needle, and the infusion cannula was introduced to a depth of 6.5 mm through the hole made at the time of tumor cell implantation. The brain infusion kit was fixed in place with surgical glue, and the pump was implanted in a subcutaneous pocket in the midscapular region, with a sufficient amount of catheter tubing to permit free motion of the animal’s head and neck. The pumps were left in place from days 7 to 13, during which time the animals received an infusion of 144 μL of carboplatin (72 μg, 194 nmol), delivered at a flow rate of 1 μL/h over 6 days, after which the pumps were removed.

The rats, were stratified into four groups and treated as follows: Group 1, Untreated controls; Group 2, Received a 6 days infusion of carboplatin (72 μg/144 μL) beginning on day 7 following tumor implantation; Group 3, Received a single 15 Gy dose of 6 MV X-rays on day 14; Group 4, Received a 6 days infusion of carboplatin, beginning on day 7 following tumor implantation in combination with a single 15 Gy dose of 6 MV X-rays administered on day 14. We have compared the survival times of these animals with the experimental groups irradiated with synchrotron X-rays tuned at 78.8 keV, as reported in detail in our previous report [12].

### Irradiation with 6 MV photons

Irradiations were performed at the University Hospital of Grenoble using a 6 MV LINAC (SLi, Elekta Oncology Systems, Ltd., West Sussex, UK). Rats were placed in a polystyrene box and were irradiated, two at a time. The head of each animal was aligned in the middle of an 8 × 4 cm^2^ aperture, defined by the beam collimator, and only the right cerebral hemisphere was irradiated. A wax block was positioned between the rats’ heads and a 0.5 cm tissue equivalent bolus was placed on top to ensure full build up of the dose at the skin surface. A dose of 15 Gy was prescribed at a 1.5 cm depth and delivered at a dose rate of 200 cGy/min (treatment planning system: Dosigray, DosiSoft, Cachan, France). After irradiations were completed, the animals were transferred to the Animal Care Facility at the ESRF. These irradiation parameters were chosen to be as close as possible to the Stereotactic synchrotron radiotherapy carried out at the European Synchrotron Radiation Facility (ESRF), which was previously described [12].

### Tumor imaging

To confirm the presence of tumor, contrast-enhanced imaging was performed after radiotherapy using a conventional CT scanner (Siemens Somatom Plus 4 Volume Zoom scanner, Siemens Medical Systems, Iselin, NJ, USA). All of the animals received an intravenous (i.v.) injection of 1.5 mL of Iomeron® (350 mg/mL of iodine), followed by 0.5 mL of a saline solution (NaCl 0.9%) via the tail vein 10 minutes before computed tomography. Four animals showed no evidence of tumor at this time and they were excluded from the therapy studies.

### Statistical methods

Kaplan-Meier survival plots were compared with the log-rank test (JMP, SAS Institute Grégy sur-Yerres, France). The log-rank test statistic compares estimates of the hazard functions of the two groups at each observed event time. It is constructed by computing the observed and expected number of events in one of the groups at each observed event time and then adding these to obtain an overall summary across all time points where there is an event. The rats’survival were considered as significantly different when p < 0.05.

## Results

### Therapeutic response following i.c. of carboplatin in combination with 6 MV X-irradiation

**Figure 1:**
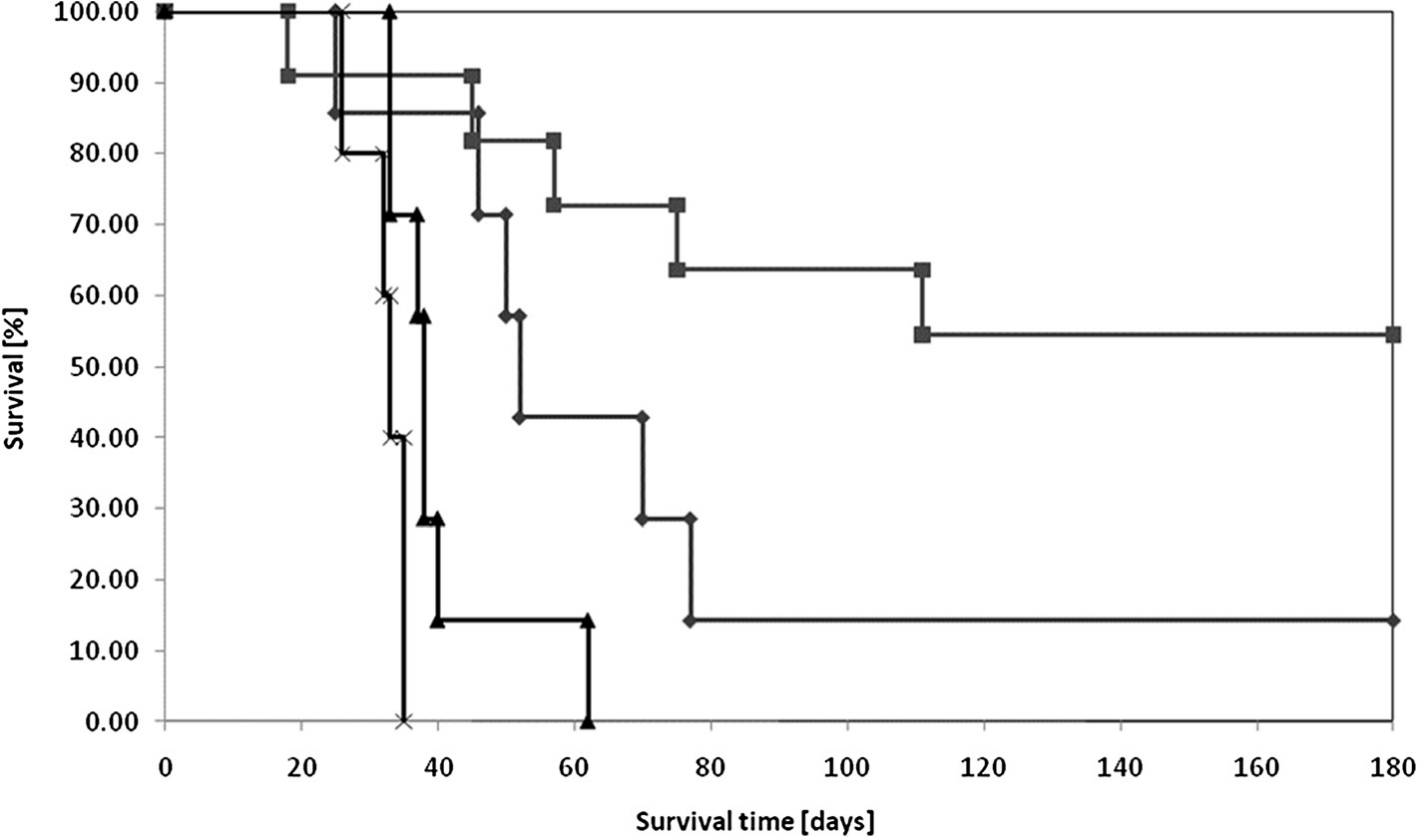
**Kaplan-Meier survival plots for glioma-bearing rats after chemoradiotherapy.** The origin of the x-axis corresponds to tumor implantation. Group 1: untreated (×); Group 2: Carboplatin alone (◆); Group 3: 6 MV X-irradiation alone (▴); Group 4: Carboplatin in combination with 6 MV X-irradiation (■).

Survival data are summarized in Table 1 and Kaplan-Meier survival plots are shown in Figure 1. The survival plots of all treatment groups were significantly different from those of untreated controls (p < 0.02). Untreated rats had a mean survival time (MST) of 32 ± 2 d compared with 40 ± 3 d for 6 MV X-irradiated animals. Rats that had received carboplatin alone had a median survival time (MeST) of 52 d and a censored MST of 71 ± 7 d, with 1 rat surviving more than 180 d, at which time the study was terminated. Animals that had received carboplatin, followed by X-irradiation with 6 MV photons, had a MST of > 126 ± 8 d and a MeST of > 180 d, with 6 of 11 rats (55%) alive at the end of the study*.* This was significantly different from irradiated animals (p <0.01) or those that had received carboplatin alone (p = 0.07).

**Table 1:** Survival times of F98 glioma-bearing rats after prolonged infusion of carboplatin with or without 6 MV X-irradiation from a LINAC source

**Group Treatment**^**a**^	**N**	**Survival time (days)**^**b**^			**% increased life span**	
		**Range**	**Mean ± SE**	**Median**	**Mean**	**Median**
Carboplatin	7	25-180 (1)^b^	71 ± 7 ^c^	52	122 ^c^	58
Carboplatin + 6MV	11	18-180 (6)^b^	126 ± 8 ^c^	>180 ^c^	294^c^	>445 ^c^
X-irradiation						
6MV X-irradiation	7	33-62	40 ± 3	38	25	15
Untreated controls	5	26-35	32 ± 2	33	-	-

^a^ Stereotactic intracerebral implantation of 10^3^ F98 glioma cells was carried out on d 0. Carboplatin (72 μg or 194 nmol) was administered over d 7–13 by means of Alzet osmotic pumps at a flow rate of 1 μL/h after which the pumps were removed.

^b^ Day 180 was the endpoint of the experiment. Rats still alive at this time were euthanized. The number in parenthesis indicates the number of rats surviving for more than 180 days (censored data).

^c^ Mean, median, or % increased life span were based on censored data.

**Figure 2:**
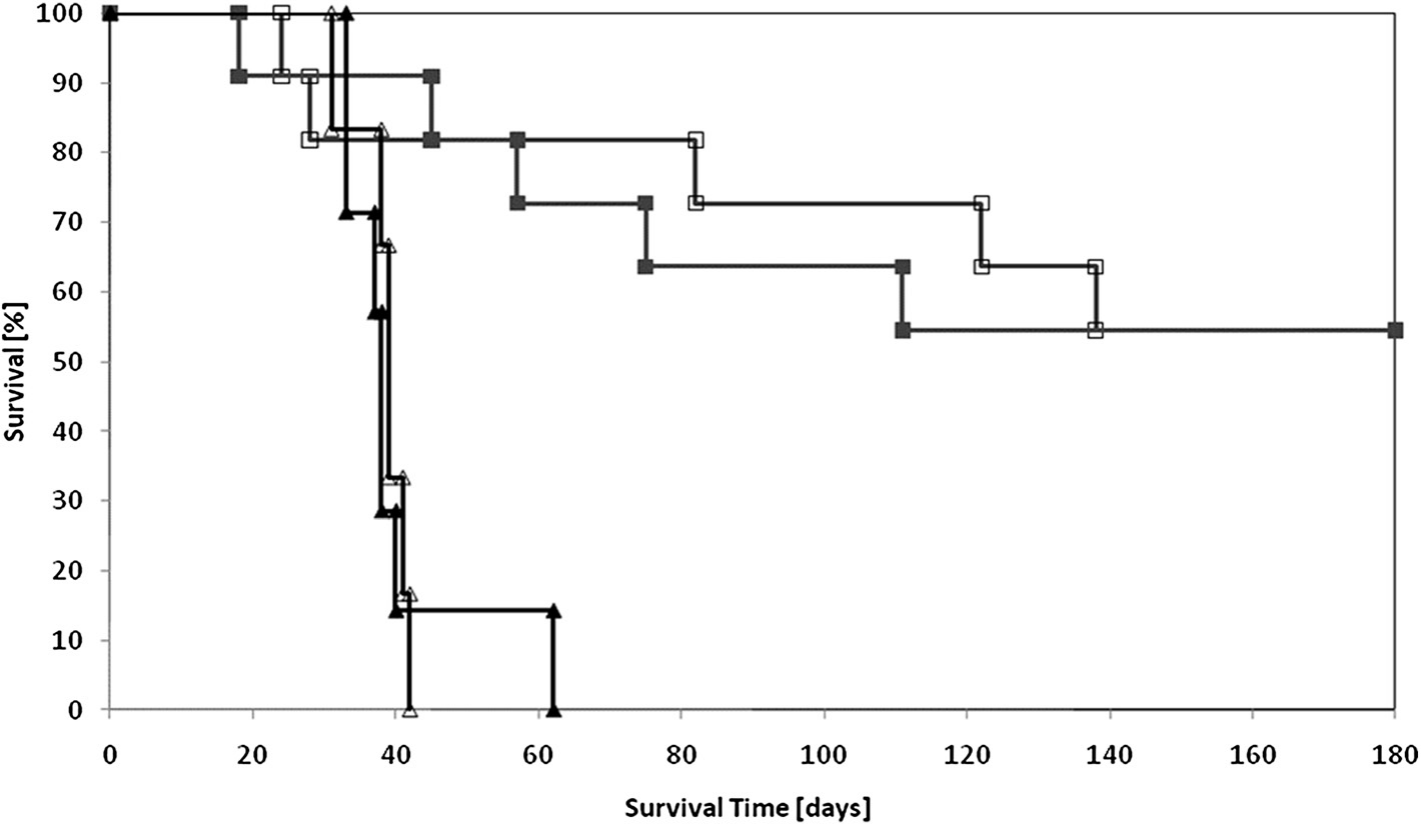
**Kaplan-Meier survival plots for glioma-bearing rats after chemoradiotherapy using either 6MV or 78.8 keV X-rays.** The origin of the x-axis corresponds to tumor implantation. Group 3: 6 MV X-irradiation alone (▴); Group 4: Carboplatin in combination with 6 MV X-irradiation (■). The empty symbols correspond to the experiments carried out at the European Synchrotron Radiation Facility in our previous study at 78.8 keV [12]. 78.8 keV synchrotron irradiation alone (Δ); Carboplatin in combination with 78.8 keV synchrotron irradiation (□).

Rats that received 6 MV photon irradiation alone or in combination with i.c. carboplatin were compared with animals that received synchrotron irradiation (data taken from our previous study [12]) (Figure 2). Although we could not repeat the synchrotron study due to an inability to schedule beam time, all the control groups (radiation alone, carboplatin alone and untreated groups) had equivalent survival times. Both radiation sources, 6 MV photons and synchrotron irradiation, resulted in equivalent survival data with p = 0.66 for the “irradiated only” groups and p = 0.88 for the “chemo-radiotherapy” groups. Similarly, equivalent survival data (p = 0.52) were observed in both experiments for those animals that received carboplatin alone (data not shown).

## Discussion

In the present study we have demonstrated that equivalent survival data were obtained in F98 glioma bearing rats that had been treated with the combination of i.c. infusion of carboplatin in combination with radiation therapy using either 6 MV photons from a LINAC or a monoenergetic beam of 78.8 keV X-rays from a synchrotron. Bernardt et al. have described the influence of relaxations of atoms attached to DNA on radiation-induced cellular DNA damage by low energy photons using Monte Carlo track structure calculations [24]. They found that the number of inner shell relaxations produced by photon irradiation generally was small in comparison to the total number of double strand breaks (DSBs) generated by the radiation itself, when the number of high Z atoms introduced by Pt containing cytotoxic drugs was small *and* compatible with cell survival studies.

Using atomic absorption spectroscopy, Guarnieri et al. and Kahn et al. have mapped the distribution of platinum after i.c. infusion of carboplatin with ALZET pumps into F98 glioma-bearing rats, with delivery parameters similar to those that we used. Platinum concentrations were maximal in brain sections corresponding to the infusion site, with diminished amounts (5 to 1 μg/g tissue) in sections that were 3 mm from the point of infusion [27,28]. The importance of the DNA damage is dependent on the number of Pt atoms intercalated with DNA molecules. At the molecular level, a larger number of DSBs were detected when cells were pretreated with cisplatin and subsequently irradiated with synchrotron X-rays above the Pt K-edge, compared to those below the K-edge [23,29]. Three times more DSBs were detected when human SQ20B squamous carcinoma cells pretreated with 30 μM cisplatin (3 ×× 10^8^ atoms of Pt atoms per cell) for 6 h [29], and 1.3 times more DSBs with the same treatment of F98 cells [23]. However, no such an enhancement was observed (even at the molecular level) with the much lower Pt concentrations that would not have been tumoricidal, when the SQ20B cells were pretreated with 3 μM cisplatin (4 × 10^6^ Pt atoms per cell) for 6 h [29]. In our studies, i.t. injection of cisplatin (3 μg in 5 μl), followed 24 h later by 15 Gy of X-irradiation, also produced similar long-term survival of F98 glioma bearing rats, *irrespective* of whether the synchrotron X-rays had energies below or above the Pt K-edge [23]. Comparable long term cure rates (17% and 18%) also were observed when the animals were irradiated with 78.8 keV synchrotron X-rays or 6 MV photons after cisplatin (6 μg in 20 μl) was administered i.c. by CED [13]. Overall, the present data and those previously reported [11-13,23,29] are in good agreement with Bernhardt et al’s. predictions [24]. They strongly suggest that the therapeutic gain obtained by the direct i.c. administration of Pt compounds, followed by X-ray irradiation, was not due to the production of Auger electrons and photoelectrons emitted from the Pt atoms, but rather involved other mechanisms. Only molecular studies performed using extremely high Pt concentrations, which were not attainable *in vivo*, demonstrated energy dependence. However, this is not an adequate explanation for the *in vivo* therapeutic efficacy of the combination of Pt based chemotherapy with X-irradiation. In order for synchrotron radiation therapy to be successful, a sufficient, but not lethal, concentration of high Z number atoms must be incorporated into or localized nearby tumor cells, to produce enough photoelectrons or Auger electrons. Other elements, such as iodine, in the form of contrast agents, or iodo-deoxyuridine (IUdR), gold, platinum or gadolinium nanoparticles are under investigations as radiation sensitizers for synchrotron stereotactic radiotherapy [30-35]. In a recent review, Kobayashi et al. [36] discussed the enhancement of radiobiological effects by heavy elements, in particular gold and platinum. Auger enhancing phenomena to electron and Hadron therapy is also suggested which broadens furthermore their therapeutic applications.

In another study [37] we have used the same chemotherapy protocol, but a different irradiation scheme: the dose was delivered in three fractions of 5 Gy using 6 MV photons and the whole brain was irradiated, beginning on the day after drug administration, using the same Alzet osmotic pumps. The results are very consis-tent with the data presented here, the chemotherapy groups had the comparable survival rates (MST of 77 d ± 23.0 and 71 d ± 7 and 16%, 14% long term survival rates, respectively). Rats bearing tumors, treated with carboplatin and X-irradiation had MST and (MeST) of 111.8 d (78 d), with 40% surviving more than 180 d (i.e. cured), compared to 77.2 d (59 d) for pump delivery of carboplatin alone and 31.8 d (32 d) for X-irradiated alone. There was no microscopic evidence of residual tumor in the brains of all long-term survivors. The biologically equivalent dose-fraction (BED) can be calculated using the classic linear quadratic equation [38,39]:

(1) $$\text{BED=n.d[1+d}{\text{.(α/β})}^{-1}]$$

where n is the number of fractions, d is the dose per fraction in Gy, and α and β are two variables that indicate the sensitivity of tumor or normal tissue to changes in dose fractionation. The α/β ratio is usually taken to be 10 for tumor and early-reacting tissues and 3 for late-reacting tissues like brain. The biologically effective dose (BED) for 15 Gy, delivered in a single fraction, using the α/β ratios indicated above, is 37.5 Gy in acute and tumor effects and 90 Gy in late effects (37). In comparison, the BEDs for 15 Gy delivered in three fractions of 5 Gy each are largely lower: 22.5 and 40.0 Gy, for tumor and normal brain, respectively. The dose per fraction should be 8 Gy, for obtaining BEDs in a three fractions regimen equivalent to those of 15 Gy delivered in a single fraction [11]. The enhanced survival results obtained using a single fraction of 15 Gy, using either 6 MV X-rays (this study) or synchrotron radiation [12], in comparison with 15 Gy delivered in 3 fractions [37] is in good agreement with the calculated equivalent BEDS of these irradiation schemes.

## Conclusions

The present study firmly establishes the equivalency of i.c. administration of carboplatin either by infusion *via* osmotic pumps or CED with irradiation with 6 MV X-rays and synchrotron X-rays. Since medical LINACs are widely available worldwide, this could provide the opportunity to clinically evaluate this combination therapy at multiple centers. Intracerebral CED of carboplatin in patients with recurrent glioblastomas (GBM) will be evaluated in a soon to be initiated Phase I at The Ohio State University. If successful, this could lead to a Phase II clinical trial evaluating the combination of i.c. of carboplatin and radiation therapy to treat patients with recurrent GBMs, for whom unfortunately there are presently no good therapeutic options.

## Competing interests

The authors declare that they have no competing interests.

## Authors' contributions

LB and HE carried out the studies and drafted the manuscript. ME, PD, JFA and FE participated to the experimental studies. JLR participated in the design of the study and in the drafting. JB participated to the irradiation and help to draft the manuscript. JR and RFB participated in the drafting. All authors read and approved the final manuscript.
